# Nasopharyngeal Carriage, Antibiotic Resistance and Serotype Distribution of Streptococcus Pneumoniae among Healthy Adolescents in Zahedan

**Published:** 2011-05-01

**Authors:** M Bokaeian, H A Khazaei, M Javadimehr

**Affiliations:** 1 Department of Laboratory Sciences, School of Paramedical Sciences, Zahedan University of Medical Sciences, Zahedan, Iran; 2 Department of Immunology and Hematology, School of Medicine, Zahedan University of Medical Sciences, Zahedan, Iran; 3 Department of Medical English, School of Medicine, Zahedan University of Medical Sciences, Zahedan, Iran

**Keywords:** Streptococcus pneumonia, Nasopharyngeal carriage, Penicillin resistance, Serotypes, Iran

## Abstract

**Background:**

Colonization of nasopharynx by Streptococcus pneumoniae can lead to pneumococcal diseases. This study was performed to determine the carriage rate of nasopharyngeal S. pneumoniae in adolescents, antibiotic susceptibility and serotype prevalence in Zahedan, Iran.

**Methods:**

Nasopharyngeal specimens from 865 adolescents (age range: 10-19 years old) attending eight schools in Zahedan, Iran, were collected and assessed by standard procedures to recover S. pneumoniae. The serotyping was carried out by latex agglutination test and the minimum inhibitory concentrations (MIC) of penicillin as well as other commonly used antibiotics were determined by a broth dilution method.

**Results:**

Pneumococci were recovered from 15.7% (136/865, 95% confidence interval (CI) 12.3-18.9) of total samples which 119 isolates were typable with the available antisera. 1, 19A, 15C, 9V, 11A and 19F were found as the most frequent serotypes. Ninety three pneumococcal isolates were sensitive to penicillin. The MIC values of antibiotics tested were (μg/ml): penicillin 0.01-4, cefotaxime 0.01-4, ceftriaxone 0.02-128, chloramphenicol 0.08-32, ciprofloxacin 0.06-16, erythromycin 0.01-128, tetracycline 0.08-128 and vancomycin 0.02-1.

**Conclusion:**

A clear diversity was seen in the serotype distribution of the S. pneumoniae isolates and most of the antibiotic resistant strains belonged to few serotypes. Healthy adolescents in Zahedan, Iran commonly show pneumococcal carriage and antibiotic resistance.

## Introduction

In community-acquired respiratory tract infections, Streptococcus pneumoniae is the most commonly isolated pathogen and it causes a variety of infections like pneumonia, acute otitis media and sinusitis.[1] Colonization of nasopharynx can lead to pneumococcal disease and hence is a potential source of horizontal spread in the community, especially in conditions of high crowding index. The highest worldwide nasopharyngeal colonization rates were reported from Africa (85-87.2%).[2] Even if nasopharyngeal isolates do not play important role in identifying the invasive disease causing agent in individuals, analysis of the dominant serotypes in a certain region reveal epidemiological importance of pneumococcal disease in the community.[3]

Distribution of invasive disease causing serotypes, colonization of nasopharynx and resistance to antibiotics could be related to age, geography, and socio-economic conditions of that population.[4][5][6][7] As the severity of the disease in this population is less, nasopharyngeal carriage and serotype distribution in adolescents have not been studied in detail. A few studies revealed pharyngeal S. pneumoniae carriage in healthy Iranian children, but pneumococcal nasopharyngeal carriage prevalence, its resistance to antibiotics and serotype distribution in adolescents are unknown. To initiate an adequate empirical antibacterial therapy, understanding of nasopharyngeal carriage of resistant Pneumococci and serotype distribution is essential. Thus we carried out this research to investigate and determine the nasopharyngeal carriage rates, serotype prevalence and in vitro antimicrobial resistance of this pathogen isolated from healthy adolescents in Zahedan, Iran.

## Materials and Methods

In this cross-sectional study, 865 healthy adolescents (aged between 10 and 19 years old: 435 females and 430 males) attending eight public schools in Zahedan, South-Eastern Iran, were registered as study samples from April 2007 to February 2008. Sample size was calculated using the formula N=Zα (2)×P(1-P)/d(2) for prevalence surveys with an expected proportion of p at 10%, an alpha of 0.05, and a level of precision (d) of 0.02. Our study protocol was approved by the Ethics Committee and the Research Council (ECRC) of the Zahedan University of Medical Sciences (ZUMS).

Nasopharyngeal samples were collected by using calcium alginate swabs and were plated immediately onto tryptic soy agar plates containing 5% sheep blood and 5 μg gentamicin ml(-1). Plates were incubated at 37˚C in 5% CO2 for 48 h. Alpha-hemolysis, optochin sensitivity, and bile solubility tests were employed to identify S. pneumoniae isolates. Pneumococcal isolates were grown in Todd-Hewitt broth plus 0.5% yeast extract and stored in 20% glycerol at -80˚C simultaneously the antibiotic susceptibility screening and serotype determination were carried out.

Pneumococcal serogroups and serotypes were determined by latex agglutination test and the Quellung reaction method using the polyclonal rabbit antisera and selected factor sera (Pneumotest-Latex kit; Statens Serum Institute, Copenhagen, Denmark) respectively.

Kirby-Bauer disk diffusion method as recommended by the National Committee for Clinical Laboratory Standards (NCCLS) M2-A6 guidelines were used to determine antimicrobial susceptibility of oxacillin (1 μg). After 24 h of incubation at 35˚C, in the presence of 5% CO2, the diameter of the zone of inhibition was measured. The inhibition zones of ≥ 20 and ≤19 mm were considered penicillin-susceptible and penicillin-resistant respectively.

Micro broth dilution method using below given concentrations of antibiotics (μg/ml) was used in order to determine the MICs of antimicrobial agents: penicillin (0.015-16), cefotaxime (0.015-8), ceftriaxone (0.02-128), chloramphenicol (1-64), ciprofloxacin (0.125-32), erythromycin (0.06-128), tetracycline (0.25-128) and vancomycin (0.5-8). Micro broth dilution method as recommended by the National Committee for Clinical Laboratory Standards (NCCLS) M7-A3 guidelines was used to determine the MICs of antimicrobial agents. A turbidity equivalent of 0.5% McFarland standard in 0.9% saline was prepared by suspending growth obtained from 5% sheep blood agar plates and diluted 1:10 to give 10(7) CFU/ml. Mueller-Hinton broth supplemented with 5% lysed horse blood containing a series of increasing concentrations of antimicrobial agents was inoculated by this dilution. The test tubes were incubated at 35˚C for 20 to 24 h and the lowest concentration of antibiotics which inhibited the visible growth of bacteria was considered as MIC. We used a penicillin-resistant strain of S. pneumoniae ATCC 49619 with a MIC of 0.25-0.5 μg/ml as control strain.

Analyses by the Chi-Square and Fisher’s Exact tests were performed in SPSS (Version 14.0 for Windows, Chicago, IL, USA). P values of <0.05 were considered significant.

## Results

Out of the total 865 adolescents (age range 10-19 years, mean 14.7±2.1), Pneumococcus was recovered from 136 samples (15.7%, 95% CI 12.3-18.9) and the pneumococcal colonization rate decreased with age (Table 1). 87.5% (119) of total isolates (136) were serotyped with the available antisera (Table 2). It was found that serotypes 1 (n=14), 19A (n=12) and 15C (n=11) were more prevalent than 9V (n=10), 11A (n=10), and 19F (n=9) serotypes (Table 2). No significant statistical difference was seen for the serotypes and age and sex (p>0.05). Because of cross reactions with more than one antisera and non availability of the antisera, 17 isolates (12.5%) could not be serotyped.

**Table 1:** Prevalence of nasopharyngeal pneumococcal colonization among adolescents by age in years (The χ(2) value for the trend is 7.34, p=0.04).

**Age (years)**	**No./total**	**Percentage (95% CI)**
10	9/35	25.7 (20.7-28.3)
11	12/47	25.5 (19.1-27.8)
12	16/78	20.5(17.7-22.9)
13	19/106	17.9 (15.5-19.8)
14	19/118	16.1 (12.6-18.4)
15	21/124	16.9 (13.1-19.6)
16	15/136	11.0 (8.7-14.4)
17	12/110	10.9 (7.2-15.8)
18	7/62	11.2 (9.5-14.3)
19	6/49	12.2 (11.1-16.7)
Total	136/865	15.7 (12.3-18.9)

**Table 2:** Distribution of serotypes of S. pneumoniae strains among adolescents. The serotypes are ranked in their order of frequency.

** Serotype **	** No. of strains **	** % **
1	14	10.29
19A	12	8.82
15C	11	8.08
9V	10	7.35
11A	10	7.35
19F	9	6.61
23F	8	5.88
23A	8	5.88
6B	7	5.14
3	6	4.41
13	5	3.67
35B	5	3.67
20	4	2.94
18C	3	2.20
10A	3	2.20
37	2	1.47
29	1	0.73
41	1	0.73
Non-typable	17	12.49
Total	136	100

Of the 136 S. pneumoniae isolates, 93 (68.5%) were penicillin-sensitive while the remaining 43 (31.5%) were penicillin-nonsusceptible (30 isolates, 22.0%, intermediately resistant and 13, 9.5%, fully resistant). The most of penicillin-resistant isolates belonged to serotypes 19A, 9V, 11A, and 23F. Table 3 shows the summary of the activities of tested antibiotics against the 136 nasopharyngeal isolates categorized by penicillin susceptibility. Based on our findings, penicillin-resistant isolates of S. pneumoniae were more likely to be resistant to other antibiotics whereas penicillin-susceptible isolates were susceptible to other tested antibiotics. 18.3% of all isolates showed full resistances to erythromycin, 9.5% to tetracycline, 9.5% to penicillin, 8.0% to chloramphenicol, 3.6% to ceftriaxone, 2.2% to cefotaxime, and 1.4% to ciprofloxacin successively. No any isolates showed resistance to vancomycin. Of the 13 isolates that were highly resistant to penicillin, 11 (84.5%) also had decreased sensitivity to chloramphenicol, 10 (76.8%) to erythromycin, 8 (61.4%) to ciprofloxacin, 7 (53.7%) to cefotaxime, 6 (46.0%) to tetracycline, and 4 (30.6%) to ceftriaxone successively. A good agreement was seen between penicillin MIC findings and oxacillin disk diffusion inhibition zone size.

**Table 3:** Sensitivity of nasopharyngeal isolates of S. pneumoniae from adolescents.

**Antibiotic**				**Penicillin sensitivity**									**MIC rang µg/ml**
****	**Total number of cases(n=136)**			**Sensitive (n=93)**			**Intermediate (n=30)**			**Resistant (n=13)**			
	**S b**	**I^c^**	**R^d^**	**S**	**I**	**R**	**S**	**I**	**R**	**S**	**I**	**R**	
Penicillin^e^	93^a^ (68.5)	30 (22.0)	13 (9.5)										<0.03-4
Cefotaxime^f^	107 (78.6)	26 (19.1)	3 (2.3)	90 (96.8)	2 (2.2)	1 (1)	18 (59.9)	8 (26.7)	4 (13.4)	6 (46.0)	4 (31.0)	3 (23.0)	0.03-4
Ceftriaxone^g^	118 (86.7)	13 (9.6)	5 (3.7)	87 (93.5)	6 (6.5)	0 (0)	20 (66.6)	9 (30.0)	1 (3.4)	9 (69.0)	1 (8.0)	3 (23.0)	<0.02-128
Chloramphenicol^h^	81 (59.5)	44 (32.4)	11 (8.1)	85 (91.4)	7 (7.6)	1 (1)	12 (40.0)	12 (40.0)	6 (20.0)	2 (15.5)	4 (31.0)	7 (53.5)	<0.08-32
Ciprofloxacin^i^	115 (84.6)	19 (13.9)	2 (1.5)	85 (91.3)	5 (5.4)	3 (3.3)	10 (33.3)	13 (43.3)	7 (23.4)	5 (38.0)	4 (31.0)	4 (31.0)	0.06-16
Erythromycin^j^	83 (61.0)	28 (20.6)	25 (18.4)	82 (88.1)	6 (6.5)	5 (5.4)	12 (40.0)	9 (30.0)	9 (30.0)	3 (23.0)	5 (38.0)	5 (38.0)	<0.01-128
Tetracycline^k^	87 (63.9)	36 (26.5)	13 (9.6)	71 (76.3)	15 (16.1)	7 (7.6)	11 (36.6)	14 (46.7)	5 (16.7)	7 (54.0)	3 (23.0)	3 (23.0)	<0.08-128
Vancomycin^l^	136 (100)	0 (0)	0 (0)	93 (100)	0 (0)	0 (0)	30 (100)	0 (0)	0 (0)	13 (100)	0 (0)	0 (0)	<0.02-1

^a^ Figures in parentheses are percentages

^b^ S=sensitive

^c^ I=intermediate

^d^ R=resistant. Break point of antibiotics (μg/ml)

^e^ penicillin=0.1

^f^ cefotaxime=32

^g^ ceftriaxone=30

^h^ chloramphenicol=16

^i^ ciprofloxacin=5

^j^ erythromycin=4

^k^ tetracycline=8

^l^ vancomycin=16.

Serotyping was available for 50 strains (86.2% of resistant strains) out of 58 pneumococcal strains that showed moderate or full resistance to at least one antibiotic class. 75.2% of the total number of resistant strains consisted of 19A, 9V, 11A and 23F serotypes.

## Discussion

On the whole nasopharyngeal carriage rate of S. pneumoniae in healthy adolescents in the present research was 15.7%. Pneumococcal nasopharyngeal carriage studies were mostly done only on children.[8] A few studies were carried on adolescents along with children or the whole population showing prevalence of pneumococcal carriage from 19 to 43%.[1][9][10] However, these investigations do not provide proper evaluation of adolescents.

In this study, the overall prevalence of penicillin full resistance (8.9%, MIC= 2 μg/ml) and penicillin intermediate resistance (22.2%, MIC=0.12-1 μg/ml) is similar to values reported worldwide ranging from 1.4 to 71%.[11] There is no sufficient information regarding antimicrobial resistance patterns of nasopharyngeal S. pneumoniae strains in healthy adolescents in Iran. The overall prevalence of penicillin resistant S. pneumoniae has been reported as 15.6% in a study carried out in Shiraz, Iran.[12] The rates of pneumococcal colonization and penicillin resistance varied greatly within regions and continents.[2]

In our study, both penicillin-resistant (38.4%) and penicillin-susceptible strains (5.3%) showed resistance to erythromycin that was similar to reports from other Asian countries.[13] For treatments of respiratory tract infections, macrolides are used as alternative of β-lactams. However lately conducted surveillance study data revealed an increase in the prevalence of macrolide-resistant S. pneumoniae in Iran and many parts of the world.[12][14] Erythromycin generally suggested as an alternative oral therapy for pneumococcal infection and penicillin-sensitive individuals. However, our results do not recommend this suggestion, since 38.4% of penicillin resistant S. pneumoniae isolates were also non-susceptible to erythromycin. Excessive consumption, inappropriate and overdose use of erythromycin and other macrolides for treatment of pneumococcal infections may be the major contributory factors for the elevated prevalence of macrolide resistance in our country and elsewhere.

It is found in our study of 43 penicillin-nonsusceptible isolates, 41.8% and 30.2% were nonsusceptible to cefotaxime and ceftriaxone respectively. The broad-spectrum cephalosporins have been suggested for treatment of pneumococcal infections, but the patients with pneumococcal meningitis from the USA and Spain did not respond at all.[15] An increase in the MIC of ceftriaxone and cefotaxime against penicillin-resistant S. pneumoniae is believed to be the cause of this incompetence. Ceftriaxone and cefotaxime are considered as alternatives in treating infections with penicillin-resistant S. pneumoniae, still their resistance to these antibiotics reveals a sign of alert.

Nasopharyngeal isolates showed high resistance to tetracycline and chloramphenicol. This resistance is possibly due to dose convenience, cost-effectiveness, easy availability over the counter and wider use of these antibiotics in the hospital as well as the community.

Eighteen multidrug resistant S. pneumoniae were isolated in our study. Resistance to penicillin and two or more non β-lactam agents such as macrolides, cotrimoxazole or tetracycline is considered as multidrug resistance. Multidrug resistant S. pneumoniae is increasingly being reported from many parts of the globe.[16] The presence or absence of a multidrug resistant phenotype can be marked by penicillin susceptibility. Strains with reduced susceptibility to penicillin usually show cross-resistance to other antibiotics and such cross-resistance was observed with erythromycin, tetracycline, chloramphenicol and ciprofloxacin. More than 75% of the antibiotic resistant strains in our study belonged to four serotypes. Katsarolis et al. and Dobay et al. reports revealed similar results.[17][18]

The increasing penicillin and multidrug resistance of S. pneumoniae bear worldwide important clinical implications. Reduced pneumococcal susceptibility in Iran may be caused by excessive antibiotic consumption and in some cases without medical prescription. The progress of pneumococcal antibiotic resistance over the last two decades has stirred up a global concern, an evolution generally related to an extensive consumption of antibiotics.[19][20] The surveillance of alteration in antibiotic susceptibilities due to time plays an important role in recognizing the potential hazards associated with S. pneumoniae infections.

In our study, most frequent serotypes were 1, 19A, 15C, 9V, 11A, and 19F in healthy adolescents. These serotypes are commonly involved in invasive pneumococcal diseases, highlighting the importance of nasopharyneal colonization in the development of serious community infections. There is no information on the nasopharyngeal carriage of S. pneumoniae and serotype distribution in healthy adolescents in Iran. In a study done by Cardozo et al. in Brazilian adolescents, it was revealed that 6B, 6A, 23F and 18C were the most common nasopharyngeal serotypes.[5] In another study done by Nascimento-Carvalho et al. in Salvador, it has been reported 14, 5, 6A, 6B, 19F, 9V, 18C and 23F as the most common serotypes.[21] In Greece on the other hand, 19F, 14, 23F and 6B serotypes have been reported as the most common pneumococcal serotypes.[22]

Considering the above findings, we can conclude that pneumococcal carriage and antibiotic resistance is common among healthy adolescents in Zahedan, Iran. A clear diversity was seen among the serotype distribution of the isolates and most of the S. pneumoniae isolates circulating in the population were invasive. Data obtained in this article and related publications emphasize the hasty need to control the proper use of antibiotics to decrease the antibiotic resistant S. pneumoniae among adolescents.

## References

[1] Bogaert D, De Groot R, Hermans PW (2004). Streptococcus pneumoniae colonization: The key to pneumococcal disease. Lancet Infect Dis.

[2] Cardozo DM, Nascimento-Carvalho CM, Souza FR, Silva NM (2006). Nasopharyngeal colonization and penicillin resistance among pneumococcal strains: a Worldwide 2004 update. Braz J Infect Dis.

[3] Brueggemann AB, Griffiths DT, Meats E, Peto T, Crook DW, Spratt BG (2003). Clonal relationship between invasive and carriage Streptococcus pneumoniae and serotype- and clone-specific differences in invasive disease potential. J Infect Dis.

[4] Aslan G, Emekdas G, Bayer M, Serin MS, Kuyucu N, Kanik A (2007). Serotype distribution of Streptococcus pneumoniae strains in the nasopharynx of healthy Turkish children. Indian J Med Res.

[5] Cardozo DM, Nascimento-Carvalho CM, Brandao MA, Azevedo GM, Ribeiro de Souza F, Silva NM, Brandao AP, Sgambatti de Andrade AL, Bradileone MC (2006). Antimicrobial resistance and serotypes of nasopharyngeal strains of Streptococcus pneumoniae in Brazilian adolescents. Microb Drug Resist.

[6] Japoni A, Kalani M, Farshad Sh, Ziyaeyan M, Alborzi A, Mehrabani D, Rafaatpour N (2010). Antibiotic-resistant Bacteria in Hospitalized Patients with Bloodstream infections: Analysis of Some Associated Factors. Iran Red Crescent Med J.

[7] Noorbakhsh Sabet N, Japoni A, Mehrabani D, Japoni S (2010). Multi-Drug Resistance Bacteria in Qom Hospitals, Central Iran. Iran Red Crescent Med J.

[8] O'Brien KL, Nohynek H (2003). World Health Organization Pneumococcal Vaccine Trials Carraige Working Group. Report from a WHO working group: standard method for detecting upper respiratory carriage of Streptococcus pneumoniae. Pediatr Infect Dis J.

[9] Hussain M, Melegaro A, Pebody RG, George R, Edmunds WJ, Talukdar R, Martin SA, Efstratiou A, Miller E (2005). A longitudinal household study of Streptococcus pneumoniae nasopharyngeal carriage in a UK setting. Epidemiol Infect.

[10] Muhlemann K, Matter HC, Tauber MG, Bodmer T (2003). Sentinel Working Group. Nationwide surveillance of nasopharyngeal Streptococcus pneumoniae isolates from children with respiratory infection, Switzerland, 1998-1999. J Infect Dis.

[11] Song JH, Jung SI, Ko Ks, Kim NY, Son JS, Chang HH, Ki HK, Oh WS, Suh JY, Peck KR, Lee NY, Yang Y, Lu Q, Chongthaleong A, Chiu CH, Lalitha MK, Perera J, Yee TT, Kumarasinghe G, Jamal F, Kamarulzman A, Parasakthi N, Van PH, Carlos C, So T, Ng TK, Shibl A (2004). High prevalence of antimicrobial resistance among clinical Streptococcus pneumoniae isolated in Asia (an ANSORP study). Antimicrob Agents Chemother.

[12] Kohanteb J, Sadeghi E (2007). Penicillinresistant Streptococcus pneumoniae in Iran. Med Princ Pract.

[13] Lee Ny, Song JH, Kim S, Peck KR, Ahn KM, Lee SI, Yang Y, Li J, Chongthaleong A, Tiengrim S, Aswapokee N, Lin TY, Wu JL, Chiu CH, Lalitha MK, Thomas K, Cherian T, Perera J, Yee TT, Jamal F, Warsa UC, Van PH, Carlos CC, Shibl AA, Jacobs MR, Appelbaum PC (2001). Carriage of antibiotic- resistant pneumococci among Asian children: a multinational surveillance by the Asian Network for Surveillance of Resistant Pathogen (ANSORP).. Clin Infect Dis.

[14] Felmingham D, Grüneberg RN (2000). The Alexander Project 1996-1997: latest susceptibility data from this international study of bacterial pathogens from community-acquired lower respiratory infections. J Antimicrob Chemother.

[15] Sloas MM, Barrett FF, Chesney PJ, English BK, Hill BC, Tenover FC, Leggiadro RJ (1992). Cephalosporin treatment failure in penicillin and cephalosporin- resistant Streptococcus pneumoniae meningitis. Pediatr Infect Dis.

[16] Lalitha MK, Pai R, Manaharan A, Appelbaum PC (2002). CMCH Pneumococcal Study Group. Multidrug resistant Streptococcus pneumoniae from India. Lancet.

[17] Katsarolis I, Poulakou G, Analitis A, Matthaiopoulou I, Roilides E, Antachopoulos C, Kafetzis DA, Daikos GL, Vorou R, Koubaniou C, Pneumatikos I, Samonis G, Syriopoulou V, Giamarellou H, Kanellakopoulou K (2009). Risk factors for nasopharyngeal carriage of drug-resistant Streptococcus pneumoniae: data from a nation-wide surveillance study in Greece. BMC Infect Dis.

[18] Dobay O, Rozgonyi F, Hajdu E, Nagy E, Knausz M, Amyes SG (2003). Antibiotic susceptibility and serotypes of Streptococcus pneumoniae isolates from Hungary. J Antimicrob Chemother.

[19] Mera RM, Miller LA, Daniels JJ, WEIL JG, White AR (2005). Increasing prevalence of multidrug-resistant Streptococcus pneumoniae in the United States over a 10-year period: Alexander Project. Diagn Microbiol Infect Dis.

[20] Baquero F, Baquero-Artigao G, Cantón R, García-Rey C (2002). Antibiotic consumption and resistance selection in Streptococcus pneumoniae. J Antimicrob Chemother.

[21] Nascimento-Carvalho CN, Freitas-Souza LS, Moreno-Carvalho OA, Alves NN, Caldas RM, Barberino MG, Duarte J, Brandao MA, Mendonca DR, Silva A, Guerra ML, Bradileone MC, Di Fabio JL (2003). Invasive pneumococcal strains isolated from children and adolescents in Salvador. J Pediatr (Rio J)..

[22] Poulakou G, Katsarolis I, Matthaiopoulou I, Tsiodras S, Kanavaki S, Hatzaki D, Roilides E, Sofianou D, Kavaliotis I, Kansouzidou A, Kafetzis DA, Paraskakis I, Foustoukou M, Daikos GL, Syriopoulou V, Pangalis A, Leveidiotou S, Giamarellou H (2007). Hellenic Study Group for the Susceptibility of Streptococcus pneumoniae. Nationwide surveillance of Streptococcus pneumoniae in Greece: patterns of resistance and serotype epidemiology. Int J Antimicrob Agents.

